# Local pressure calibration method of inductively coupled plasma generator based on laser Thomson scattering measurement

**DOI:** 10.1038/s41598-022-08679-y

**Published:** 2022-03-18

**Authors:** Jinhai Sun, Yong-Qiang Liu, Yan Zheng, Jielin Shi, Yu Li, Yarui Zhao, Xutao Zhang, He Cai, Xianli Zhu, Xinxue Sun, Zengming Chao, Hongcheng Yin, Hongbin Ding

**Affiliations:** 1 Science and Technology on Electromagnetic Scattering Laboratory, Beijing, 100854 China; 2 School of Physics, Dalian University of Technology, Dalian, 116024 China

**Keywords:** Plasma physics, Magnetically confined plasmas

## Abstract

Based on laser Thomson scattering (TS) measurements and finite element method (FEM) simulations of electron density in inductively coupled plasma (ICP), the simulated local pressure calibration curves of ICP generator are obtained by comparing the experimental and simulated electron density distributions and maxima. The equation coefficients of theoretical model associated with the ICP generator experimental system can be obtained by fitting the simulation curve with the least square method, and the theoretical pressure calibration curves under different absorbed powers can be further obtained. Combined with the vacuum gauge measurements, both the simulated and theoretical pressure calibration curves can give the true local pressure in the plasma. The results of the local pressure calibration at the different absorbed powers show that the density gradient from the vacuum gauge sensor to the center of the coil in ICP generator cavity becomes larger with the increase of electron density, resulting in a larger gap between the measured value and the pressure calibration value. This calibration method helps to grasp the local pressure of ICP as an external control factor and helps to study the physicochemical mechanism of ICP in order to achieve higher performance in ICP etching, material modification, etc.

## Introduction

Inductively Coupled Plasma (ICP) has been widely used for crystal etching^1^, semiconductor etching^2^, thin film deposition^3^, material surface modification^4,5^ and so on. However, accurate and skilled use of these processes requires a clear understanding of the various ICP control factors such as ICP internal pressure and absorbed power. These control factors can sensitively affect the electron density, electron temperature and other physical parameters of ICP, thus profoundly affect the accuracy of production process. Especially important, the local pressure in a low-pressure plasma affects the mean free path of collisions between electrons and neutral particles, which is closely related to local and non-local dynamic characteristics^6^, electron heating and heating efficiency, electron energy distribution^7^ and electron density^8^, discharge mode (E or H mode)^9,10^ and so on. Therefore, the local pressure is a very important external parameter of low-pressure plasma, especially in terms of accuracy control during the plasma process. As an electrical parameter, absorbed power can be accurately measured by tracing its source. However, the local pressure in ICP is difficult to be accurately measured because of the distance between the sensor of the vacuum gauge and the plasma region to be measured. To understand the actual pressure in the plasma, it is necessary to calibrate the local pressure in ICP. This provides quantitative pressure parameters for studying the physicochemical mechanisms of various processes in ICP.

Electron density and electron temperature are the main physical parameters to characterize the plasma, so the high agreement between their finite element simulation results with the experimental results can fully guarantee the reliability of the computational model. Both simulation and experiment reveal that the gradient in the electron density distribution of ICP is very pronounced under H-mode discharge condition. The plasma is firmly bound inside of the coil by the inductor coil. As a statistical physical quantity, the measured or simulated electron temperature has no significant gradient distribution. Obviously, the identification of electron density is much higher than that of electron temperature^11,12^ and there is a close correlation between electron density and electron temperature^13^. So we can focus on comparing the distribution of electron density to determine whether the external conditions of the simulations and experiments in this paper are consistent. However, this comparison depends on accurate and reliable measurements of electron density. At present, the commonly used measurement methods for ICP electron density include Langmuir probe method^14^, emission spectroscopy^15^, microwave interferometry^16^, laser interferometry^17^ and laser Thomson scattering (TS) measurement^18^. Among these techniques for measuring plasma electron density, the TS can measure the electron density in a given region without disturbing the plasma, and has very high spatial and temporal resolution^19^. It is recognized as one of the most accurate electron density measurements available. So in this paper we use TS to measure the electron density distribution of ICP.

Accurate measurement and calibration of pressure, especially local pressure, has rarely been reported in previous research work. In 2007, Shimada^20^ measured the spatial distribution of neutral gas temperature and total pressure, electron pressure and neutral pressure by inserting a thin tube probe with diameter of 3.15 mm into the plasma. However, this interventional measurement itself interferes with the local environment of the measurement area, making it difficult to achieve accurate measurements. In order to avoid the measurement uncertainty caused by interventional measurements, a new method of ICP local pressure calibration combining TS experiments and finite element method (FEM) simulations is proposed in this paper. First, the electron density distribution of ICP is measured non-invasively by laser TS. The ICP pressure to be calibrated is measured by a vacuum gauge probe (TPG 201, Pfeiffer Vacuum) at the gas inlet of the ICP generator. The absorbed power of the ICP is the product of the input power of a 13.56 MHz RF power supply and the power transfer efficiency. As the power and pressure change, the plasma density changes, and the plasma load on the matching network varies accordingly. To compare the experimental and simulated values of electron density distribution at a specific absorbed power, the capacitance in the matching circuit is experimentally adjusted by Smith chart to match the internal impedance of the RF power supply so that a given power such as 400 W, 350 W and 300 W is transmitted into the plasma. The power transfer efficiency is 96.3%, 94.9% and 93.0% at an absorbed power of 400 W, 350 W and 300 W, respectively, as measured by a similar method in reference^21^. Second, a FEM model with the same physical dimensions as the ICP generator experimental setup is established. At the same absorbed power as the experiment, the pressure measured by the vacuum gauge is taken as the reference starting point, and the pressure is scanned in the lower pressure direction. The resulting simulation yields a series of spatial distributions of the electron density with pressure in the ICP generator cavity. Third, this series of plasma electron density simulation data is used as the true value of the measurement and compared with the electron density data measured by TS. When both the experimental and simulated electron density distributions and maxima are within the error tolerance, the pressure value in the simulation can be determined as the corrected value for the pressure measurement. This completes a single pressure data calibration of the ICP generator. This cycle can be repeated to complete a pressure calibration simulation curve for a specific ionized gas at a given absorbed power. At last, combined with the theoretical model, the equation coefficients associated with the ICP generator experimental system are obtained by fitting the pressure calibration simulation curve using the least square method, so as to obtain the theoretical pressure calibration curves for the different absorbed powers. Both simulated and theoretical calibration curves can be used for the local pressure calibration in low-pressure plasma processes.

## Laser TS experiment

Laser TS technique can measure the electron density and electron temperature of plasma by measuring the secondary radiation emitted by the interaction between free electrons and the incident laser^22^. When a laser with a wavelength of *λ*_0_ enters plasma, the free electrons in the plasma radiate electromagnetic waves under the action of the incident laser electric field. Since TS is elastic scattering, the wavelength of the scattered light is the same as that of the incident laser. However, the Doppler Effect is obvious due to the fast electron motion, so the Doppler broadening of TS spectrum appears. The electron density and electron temperature of plasma can be obtained by measuring TS spectrum and data post-processing.

**Figure 1:**
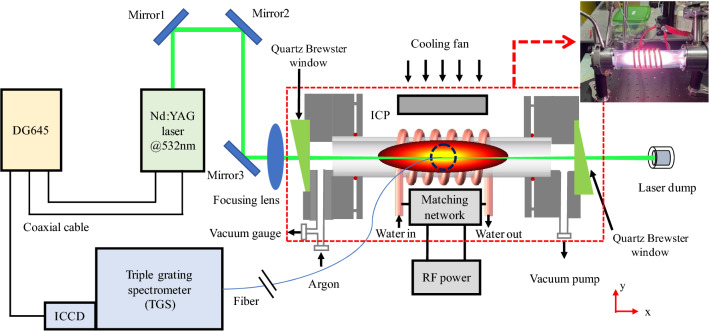
Schematic diagram of the TS experiment. In the upper right corner is a photo of the ICP generator corresponding to the red box in the schematic.

As shown in Fig. 1, the ICP generator is placed in our TS experimental system, using Nd: YAG's double frequency of 532 nm laser with repeat frequency of 30 Hz, maximum laser pulse energy of 300 mJ, pulse width of 10 ns as detection light, and triple-grating spectrometer (TGS) as a detection system. The laser is transmitted to the window of the plasma vacuum cavity through some high reflection mirrors dedicated for high intensity laser. To avoid the influence of stray light on TS signal, Brewster windows are adopted in the incident window and the exit window. The laser entering the ICP generator cavity is focused to the center of the plasma beam by a focusing lens. The laser passing through the plasma eventually enters the laser dump and terminates transmission.

In order to keep the flexibility of space, we use optical fiber to collect the TS signal and transmit them to the slit of TGS system in this scheme. The collection direction of optical fiber is at a 90-degree angle to the laser transmission direction and the axial direction of the plasma beam. A set of plano-convex lenses is arranged in front of the fiber to focus the laser-plasma interaction region to the fiber entrance. To avoid Rayleigh scattering from the air and reflections from other surfaces, the collection end of the optical fiber and the collection lens are placed in a dark chamber. The TS signal is transmitted to the TGS system through the optical fiber, passing through the grating, lens and other optical devices in the TGS system, and finally is imaged or formed into spectrum on the ICCD camera. The DG645 is a time controller that synchronizes the laser pulse with the ICCD camera shutter and optimizes the detected signal by changing the ICCD camera exposure time. The TGS system can effectively filter out Rayleigh scattering and stray light, which is beneficial to the extraction of laser TS signal. The electron density measured with TS in this experiment ranges from 10^18^ m^-3^ ~ 10^19^ m^-3^.

**Figure 2:**
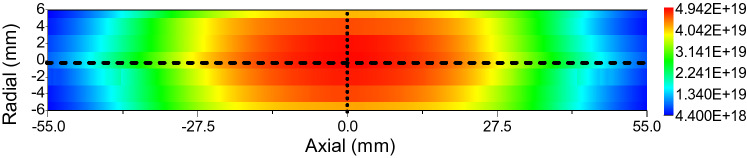
Electron density distribution measured by laser TS at 350 W absorbed power and 65 Pa pressure. The dotted line and dashed line in the figure indicate the locations of the cut line data to be collected.

The electron density distribution of ICP measured with laser TS at 350 W absorbed power and 65 Pa pressure (measured by vacuum gauge) is shown below (Fig. 2).

Experimental parameters such as ICP pressure and absorbed power were recorded during the experiment. The pressure measured in the experiment was used as the reference starting value of the pressure parameter scanning in the plasma simulation. The parameters such as absorbed power, coil size, and cavity dimensions were kept consistent with the experimental system when the ICP was simulated.

## FEM simulation

The Frequency-Transient study is used to simulate ICP with COMSOL software in this paper. The diffusion model is Mixture-averaged including migration in electric field. Reduced electron transport characteristics with local energy approximation are used in plasma properties settings. The electron energy distribution function is set as Maxwellian. And the model temperature is specified as 300 K. The electron transport property is restricted to specify mobility only, and the isotropic reduced electron mobility is set to 4 × 10^24^ (V m s)^−1^.

**Figure 3:**
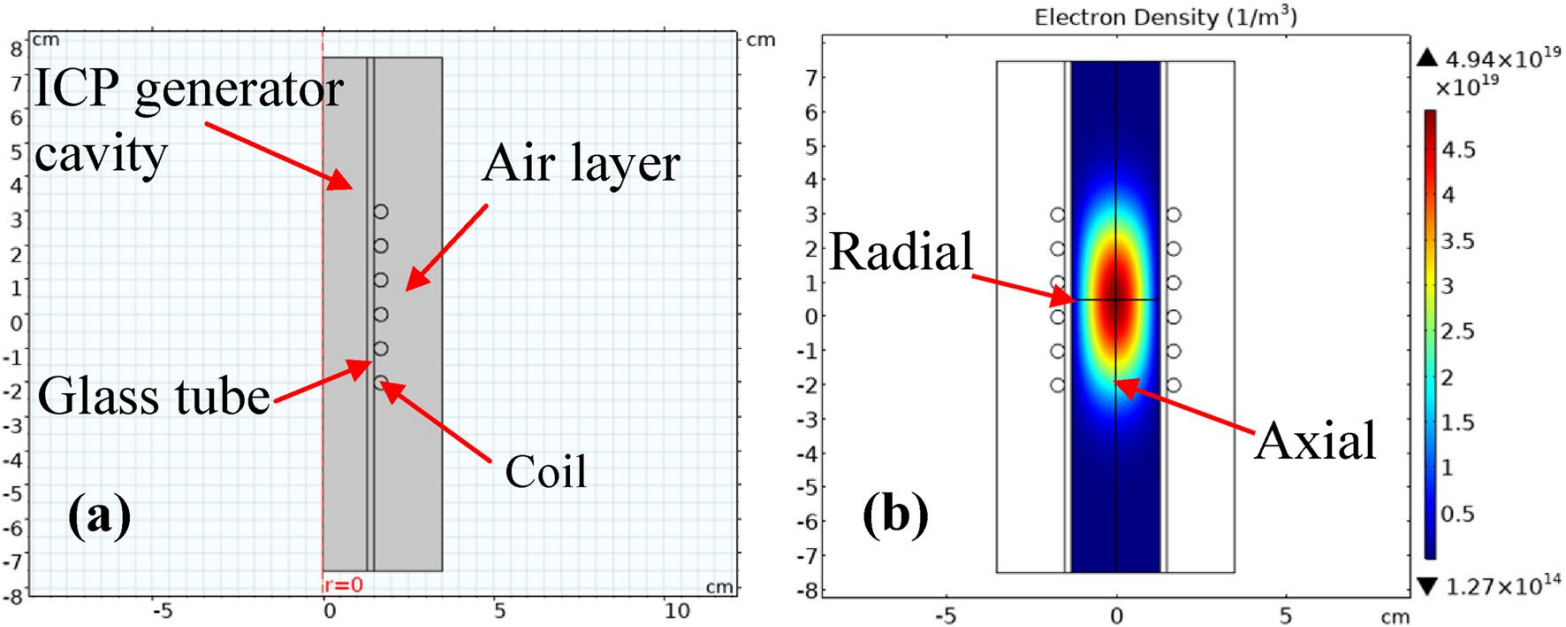
(**a**) ICP simulation model schematic diagram and (**b**) electron density distribution. The radial and axial lines in (**b**) indicate the location of the cut line data to be collected.

The finite element model of ICP is developed according to the physical dimensions of its generator experimental system. Since the axisymmetric tubular structure is to be simulated, a 2D axisymmetric simulation model is established to save computational resources, as shown in Fig. 3a. The length of the tube is 15 cm. The inner diameter is 2.6 cm. The wall thickness is 0.2 cm. The coil has 6 turns, the wire diameter of the coil is 0.4 cm and the spacing between turns is 1 cm. The thickness of the air layer outside the tube is set as 2 cm. The tube is filled with argon gas to be ionized. For the ionized argon gas, there are seven kinds of electrochemical reactions and two kinds of surface reactions on the tube wall. The types and coefficients of electrochemical reactions are shown in Table 1, and the surface reactions on the tube wall are shown in Table 2.

**Table 1:** Electrochemical reactions in an argon ionized plasma.

	Reaction equation	Type	ΔE (eV)
1	e + Ar = > e + Ar	Elastic collision	0
2	e + Ar = > e + Ars	Excitation	11.5 V
3	e + Ars = > e + Ar	Superelastic collision	− 11.5 V
4	e + Ar = > 2e + Ar +	Ionization	15.8 V
5	e + Ars = > 2e + Ar +	Ionization	4.427 V
6	Ars + Ars = > e + Ar + Ar +	Penning ionization	–
7	Ars + Ar = > Ar + Ar	Metastable quenching	–

**Table 2:** Electrochemical reactions on the inner surface of the tube wall.

	Reaction equation	Adhesion coefficient
1	Ars = > Ar	1
2	Ar + = > Ar	1

Convective flux is not considered and therefore no gas inflow and outflow are used. The air surrounding the coil is considered to possess vacuum characteristics. During the simulation, the electron neutral initial value of argon ion and the neutral mass of argon atom are constrained. The wall surrounding the plasma is grounded and the general wall reflection coefficient is set as 0.2. The initial electron density is set as 1 × 10^15^ m^−3^, and the initial average electron energy is set as 5 V. The coil is set to a single wire model in the magnetic field settings, specifying the absorbed power. The model is filled with triangle meshes of 17,356 cells and the minimum cell is set to 45 µm. Boundary layers have been created next to the walls in order to solve properly the high gradients formed during the simulation. Each boundary layer is divided into 5 sub-layers and smoothed over to the interior mesh. The mesh has been refined several times. The external boundaries of the whole geometry are magnetically insulated excepting the symmetry axis.

When the ICP device is first energized, all the power dissipated is in the coil. After about 1 µs, the plasma ignition begins and as the neutral gas atoms split into electrons and ions, the electrons begin to absorb more and more power and further ionize the neutral gas. The local electron density in the center reaches instantaneous maximum value in more than 30 µs, and then reaches a stable state with the diffusion of electrons. In order to ensure that the ICP could reach a stable state, the calculation time should be set long enough. The electron density distribution evolution over time varies with pressure and absorbed power, but 0.1 s is sufficient to achieve a stable distribution in our study. In order to understand the evolution process of ionization over time and ensure that the electron density distribution reaches a stable state, we have performed simulations with 20 time interpolation points between 10^–6^ s and 10^–1^ s.

## Results and discussion

**Figure 4:**
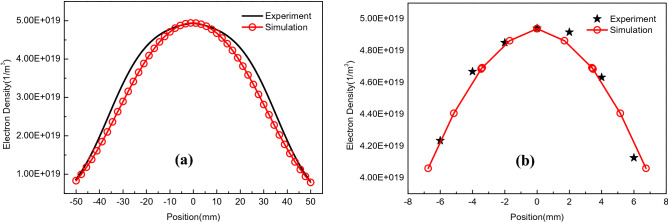
Comparison of experimental and simulated data of the axial electron density distribution (**a**) and the radial electron density distribution (**b**) at the center of ICP.

The analysis of the plasma electron density distribution was carried out after convergence. As the plasma electron density is axisymmetric, cut line data should be collected along the axis of the glass tube and perpendicular to the axis of the glass tube at the center of the coil as shown in Fig. 3b. 2D drawings of the axial and radial cut line data at the center of the coil are plotted in Fig. 4. And they are compared with the experimental data obtained from TS measurement at corresponding positions respectively as shown in Fig. 2. Thus, the consistency between the simulated data and experimental data is used to determine which pressure value in the simulation is the calibration value of the measured value.

**Figure 5:**
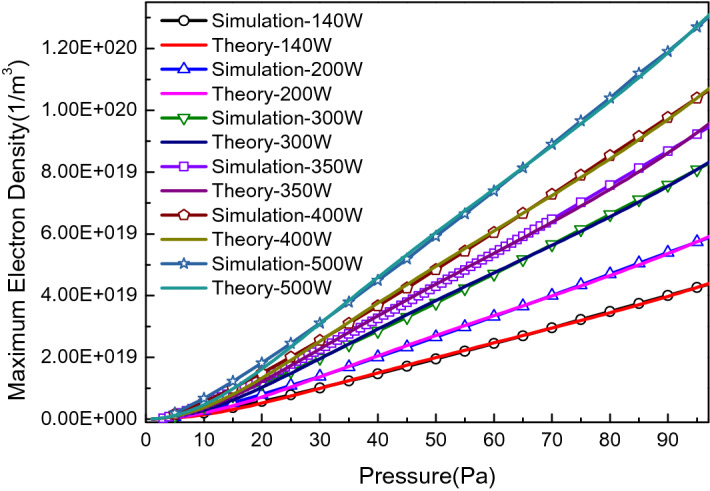
Relationship between maximum electron density and pressure at different absorbed powers.

During the simulation, a series of electron density distribution data for a certain absorbed power can be obtained by scanning the pressure parameters. Capturing their maximum values and plotting them with a series of pressure-related dotted lines, a local pressure correction curve for the ICP center can be obtained, as shown in Fig. 5. Since these pressure calibration simulation curves vary with absorbed power, a unified theoretical model is needed to facilitate the depiction of pressure calibration curves in future experimental work.

The dynamic behavior of the electron density is characterized by the ambipolar diffusion of electrons and ions. The plasma-electron rate equation based on the ambipolar diffusion is obtained from the continuity equation^23^

1 $$\frac{\partial n_{e} }{\partial t} - D_{A} \nabla^{2} n_e = Q\left( {r,z,t} \right),$$

where *D*_*A*_ is the ambipolar diffusion coefficient and *n*_*e*_ is the electron density. In cold plasmas like ICP, $D_{A} = kT_{e} /M\upsilon_{c}$ where *k* is Planck's constant, *T*_*e*_ is the electron temperature of plasma, *M* is the rest-mass of ions^24^. And $\upsilon_{c}$ is the ion-neutral collision frequency which is proportional to the density of neutral particle $n_{g}$ expressed as $\upsilon_{c} = n_{g} \sigma_{i} \left( {kT_{i} /M} \right)^{1/2}$, where $\sigma_{i}$ is the collision cross section of ion to neutral and *T*_*i*_ is the ion temperature. The right term *Q*(*r, z, t*) in Eq. (1) is the source of plasma generation by the RF power, where electrons basically ionize neutrals by collision. So the source term is expressed as $Q\left( {r,z,t} \right) = n_{e} n_{g} K_{i} \varepsilon_{i}$, where *K*_*i*_ is the average ionization rate coefficient, including the ionization of the ground state and several excited states. $\varepsilon_{i}$ is the total ionization energy. In the steady-state case characterized by $\partial n_{e} /\partial t = 0$, the diffusion loss of the plasma may be in balance with the generation from the plasma source. Then, the Eq. (1) is simplified to

2 $$n_{g} K_{i} \varepsilon_{i} = \sqrt {\frac{k}{{MT_{i} }}} \frac{{T_{e} }}{{\Lambda^{2} \sigma_{i} n_{g} }},$$

where the symbol $\Lambda = 1/\left( {\partial /\partial r} \right)$ represents the inverse of the density gradient, which depends sensitively on the geometrical configuration and other physical conditions in the individual experiment. The electrons in the plasma will move inside the discharge tube. The mean free path $\lambda$ of electron in the discharge tube is inversely proportional to the product of the electron scattering cross section $\sigma_{e}$ and the neutral particle density $n_{g}$ expressed as $\lambda = 1{/}\left( {\sigma_{e} n_{g} } \right)$. The electrons inside the discharge tube are accelerated by the induced and residual electric field $E = E_{in} + E_{re}$, gaining their kinetic energy of $\lambda eE$ before they collide with neutrals. The induced electric field $E_{in}$ is produced by the time variation of the magnetic field caused by the coil current of the RF power supply. It is an important electron-heating source under high pressure. The residual electric field $E_{re}$ is caused by ambipolar diffusion, eventually leading to plasma potential, which may be an important process of electron-heating at low pressure^25^. Electrons scatter isotropically in collisions with neutral particles, thermalizing the energy they gain. This process is repeated until their temperature *T*_*e*_ is determined. Thus, the electron temperature is proportional to the product of the mean free path and the total electric field inside the discharge tube^26^ expressed as

3 $$T_{e} = \xi \lambda eE = \frac{\xi eE}{{\sigma_{e} n_{g} }},$$

where *ξ* is the thermalization form factor of electron energy. Substituting Eq. (3) into Eq. (2), we get^24^

4 $$n_{g} K_{i} \varepsilon_{i} = \sqrt {\frac{k}{{MT_{i} }}} \frac{\xi eE}{{\Lambda^{2} \sigma_{e} \sigma_{i} }}\frac{1}{{n_{g}^{2} }}.$$

We assume that $P_{in}$ is the power density inside the discharge tube provided by the induction coil from the RF power system. The power balance equation is given by^27^

5 $$P_{in} = n_{e} n_{g} K_{i} \varepsilon_{i} + n_{e} n_{g} K_{ex} \varepsilon_{ex} + n_{e} n_{g} K_{c} \frac{{3m_{e} }}{M}T_{e} .$$

where $K_{ex}$ and $K_{c}$ are the average excitation and collision rate coefficients respectively, $\varepsilon_{ex}$ is the average excitation energy, and $m_{e}$ is the electron mass. Substituting Eq. (3) and Eq. (4) into Eq. (5), we get

6 $$P_{in} = n_{e} \left( {\sqrt {\frac{k}{{MT_{i} }}} \frac{\xi eE}{{\Lambda^{2} \sigma_{e} \sigma_{i} }}\frac{1}{{n_{g}^{2} }}{ + }n_{g} K_{ex} \varepsilon_{ex} + K_{c} \frac{{3m_{e} }}{M}\frac{\xi eE}{{\sigma_{e} }}} \right).$$

For the present experimental conditions, the neutral density $n_{g}$ is much higher than the electron density $n_{e}$. So we expect that the neutral density $n_{g}$ is linearly proportional to the pressure $p$ expressed as $n_{g} = sp$, where $s$ is the proportional coefficient. Then we get

7 $$n_{e} = \frac{{P_{in} }}{{\sqrt {\frac{k}{{MT_{i} }}} \frac{\xi eE}{{\Lambda^{2} \sigma_{e} \sigma_{i} }}\frac{1}{{s^{2} p^{2} }} + sK_{ex} \varepsilon_{ex} p{ + }K_{c} \frac{{3m_{e} }}{M}\frac{\xi eE}{{\sigma_{e} }}}} = \frac{{P_{in} }}{{A + Bp^{3} + Cp^{2} }}p^{2} .$$

where $A = \sqrt {\frac{k}{{MT_{i} }}} \frac{\xi eE}{{\Lambda^{2} \sigma_{e} \sigma_{i} }}\frac{1}{{s^{2} }}$, $B = sK_{ex} \varepsilon_{ex}$ and $C = K_{c} \frac{{3m_{e} }}{M}\frac{\xi eE}{{\sigma_{e} }}$. *A* and *C* are directly proportional to the total electric field *E*. As shown in Table 3, the same theoretical model has different parameters under the different absorbed powers, corresponding to different relationship curves between maximum electron density and pressure, as shown in Fig. 5.

**Table 3:** Theoretical model parameters for different absorbed powers.

	Absorbed power (W)	A	B	C
1	140	9.21E−15	− 2.41E−20	4.55E−18
2	200	9.40E−15	− 2.62E−20	4.93E−18
3	300	9.78E−15	− 2.83E−20	5.31E−18
4	350	9.46E−15	− 3.34E−20	5.90E−18
5	400	1.01E−14	− 2.93E−20	5.52E−18
6	500	1.03E−14	− 3.05E−20	5.69E−18

**Figure 6:**
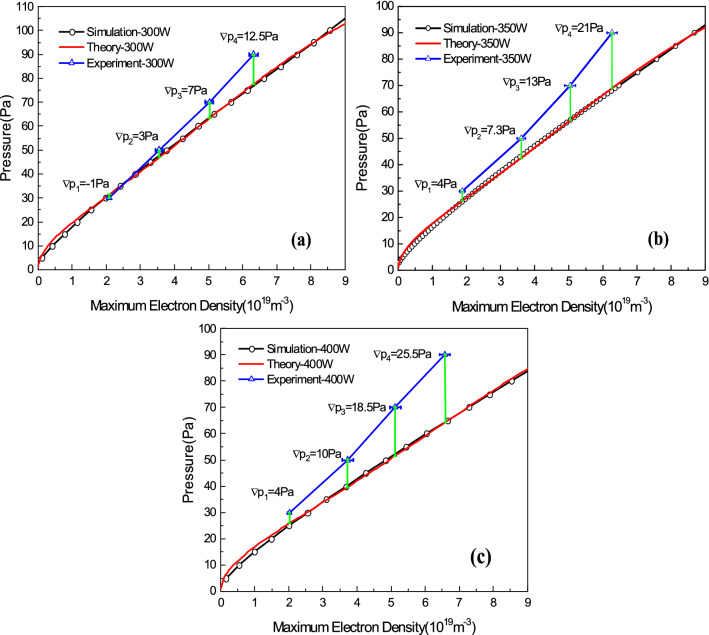
Pressure calibrations at 300 W (**a**), 350 W (**b**) and 400 W (**c**) absorbed powers.

Taking three absorbed powers of 300 W, 350 W and 400 W as examples, the relationships between the maximum electron densities measured by laser TS and the pressures measured by vacuum gauge have been compared with the simulated and theoretical results, as shown in Fig. 6. It can be clearly seen that at the same maximum electron density, the pressure measured by the vacuum gauge deviates from the simulated and theoretical matching pressure values, which is the significance of pressure calibration. From the deviation comparison in Fig. 6, we know that the measurement deviation of the vacuum gauge increases with increasing maximum electron density, which means that the pressure gradient from the vacuum gauge sensor to the center of the coil in ICP generator cavity becomes larger as a consequence.

The pressure difference between the experimental and calibrated values shown in Fig. 6 is actually due to the pressure gradient at two different locations, the vacuum gauge sensor and the center of the coil in ICP generator cavity. Since the pressure *p* and the density *n* can be related by a thermodynamic equation $p = nkT$, their gradient relationship is $\nabla p = \nabla nkT$ where *T* is the temperature of the particle^23,27^. We can assume that the neutral density on the vacuum gauge sensor $n_{gs}$ is equal to the sum of the neutral density $n_{gc}$ and the ion density $n_{i}$ at the center of the coil $n_{gs} = n_{gc} + n_{i}$. And the ion temperature is equal to the temperature of gas $T_{i} \approx T_{g}$ for the cold plasma of ICP. Because the pressure at the center of the coil in ICP generator cavity is $p_{c} = n_{gc} kT_{g} + n_{i} kT_{i} + n_{e} kT_{e}$ and the pressure on vacuum gauge sensor is $p_{s} = n_{gs} kT_{g}$, we can get the pressure gradient at these two different positions $\nabla p = p_{c} - p_{s} \approx n_{e} kT_{e}$. It can be seen that with the increase of electron density, the density gradient from the vacuum gauge sensor to the center of the coil in ICP generator cavity will also become larger, resulting in a larger gap between the measurement value and the calibrated value of pressure.

## Conclusions

In this paper, the plasma electron density distributions of ICP are obtained by means of TS experiment and FEM simulation. The simulated calibration value for the pressure measured by the vacuum gauge has been determined by comparing simulated and experimental results of the electron density distribution and the maximum electron density under the same conditions including the absorbed power. As a result, calibration simulation curve has been obtained for a given absorbed power. A more accurate theoretical equation for the relationship between maximum electron density and pressure has been derived for the first time. And the simulation curves have been matched with the theoretically derived equation to obtain the exact equation parameters for given powers. As calibration examples, three theoretical fitting curves have been used to calibrate the pressures at 300 W, 350 W and 400 W respectively for the same ICP generator. The pressure calibration results show that the pressure gradient from the vacuum gauge sensor to the center of the coil in ICP generator cavity become larger with the increase of electron density. The control of the local pressure at the ICP center point will help to improve the accuracy of low-pressure plasma processes such as plasma thin film deposition, etching and material surface treatment in the future.
